# Composites Additive Manufacturing for Space Applications: A Review

**DOI:** 10.3390/ma15134709

**Published:** 2022-07-05

**Authors:** Sung Wook Paek, Sivagaminathan Balasubramanian, David Stupples

**Affiliations:** 1 School of Science & Technology, City University London, Northampton Square, London EC1V 0HB, UK; d.w.stupples@city.ac.uk; 2 Samsung R&D Institute India, Karnataka 560037, India; siva4u@gmail.com

**Keywords:** additive manufacturing, composites, structures, thermal protection, radiation shields, electronics, satellites, spacecraft

## Abstract

The assembly of 3D printed composites has a wide range of applications for ground preparation of space systems, in-orbit manufacturing, or even in-situ resource utilisation on planetary surfaces. The recent developments in composites additive manufacturing (AM) technologies include indoor experimentation on the International Space Station, and technological demonstrations will follow using satellite platforms on the Low Earth Orbits (LEOs) in the next few years. This review paper surveys AM technologies for varied off-Earth purposes where components or tools made of composite materials become necessary: mechanical, electrical, electrochemical and medical applications. Recommendations are also made on how to utilize AM technologies developed for ground applications, both commercial-off-the-shelf (COTS) and laboratory-based, to reduce development costs and promote sustainability.

## 1. Introduction

Additive manufacturing (AM) has recently adopted a wide variety of materials ranging from plastics, metals, their composites, and alloys. Food, fabric, concrete, and cement are amongst the new printable materials that have been considered commercially. The application areas of AM have also ever been expanding toward the field of space engineering beyond terrestrial applications. Exploring diverse materials and applications will achieve the short-term goal of affordable access to space and the long-term goal of mankind’s prolonged existence beyond Earth. In the near term, 3D-printed structures currently prepared on Earth will be manufactured or/and assembled on low-Earth orbit (LEO); both on-Earth and off-Earth AM activities are expected to enhance the logistics and supply chain management of spare parts for space missions [1,2,3,4]. The long-term goal of establishing space habitats in on the Moon or Mars will require 3D printing of nearly all printable materials aforementioned to independently sustain astronauts’ lives through structural, electrical, and biomedical applications similar to those on Earth.

SpaceX first used a 3D-printed component in 2014, which was a liquid oxygen valve in one of the nine engines inside the Falcon 9 rocket; currently, an entire engine of a small rocket can be 3D manufactured using large-scale metal printers with robotic arms, which reduce the number of assembled engine parts by an order of magnitude [5,6]. These 3D metal printers are heavy and energy-intensive because of their laser melting process, as is compared with composite 3D printing in the last section. Composite 3D printers, on the other hand, suffer less from these constraints because composites have much lower melting points than metals. Composite 3D printers have been installed in the International Space Station (ISS) for astronauts’ use. Different versions and brands of commercial 3D printers have proved their functionality under microgravity. However, they are not currently being used outside the ISS due to harsh space conditions such as vacuum, radiation, micrometeorites, etc. The first automated on-orbit AM will be demonstrated in the near future to produce structural components with large dimensions and aspect ratios. Considering this background and context, the latest development of AM technologies for composite manufacturing and assembling is discussed within mechanical, electrical, and medical applications in space.

## 2. 3D-Printed Structures

Mechanical components and structures are one of the space applications wherein AM can be applied almost immediately due to their high technology readiness level (TRL). Those 3D-printed components exhibit mechanical performance comparable to conventionally manufactured space structures; they can also be functionalised during the AM process to possess shielding capabilities against heat and radiation. After the ongoing technological demonstrations of AM onboard the ISS, the next envisioned step would be to utilise AM for servicing, assembling, and manufacturing in space. One of such efforts is the 2022 NASA mission where a satellite will autonomously manufacture deployable composite booms on orbit using its onboard 3D printer.

### 2.1. Heat Shields for Suborbital Flight

**Figure 1:**
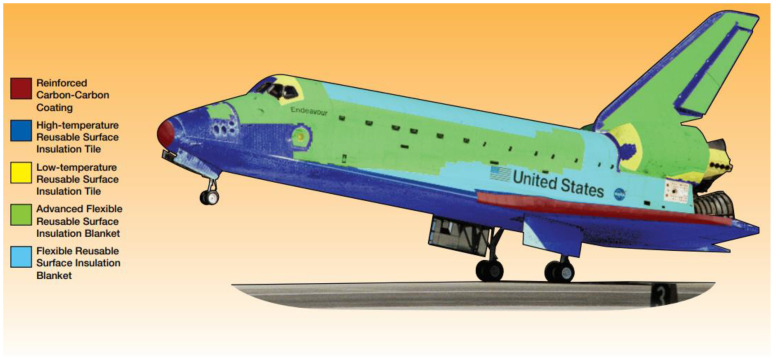

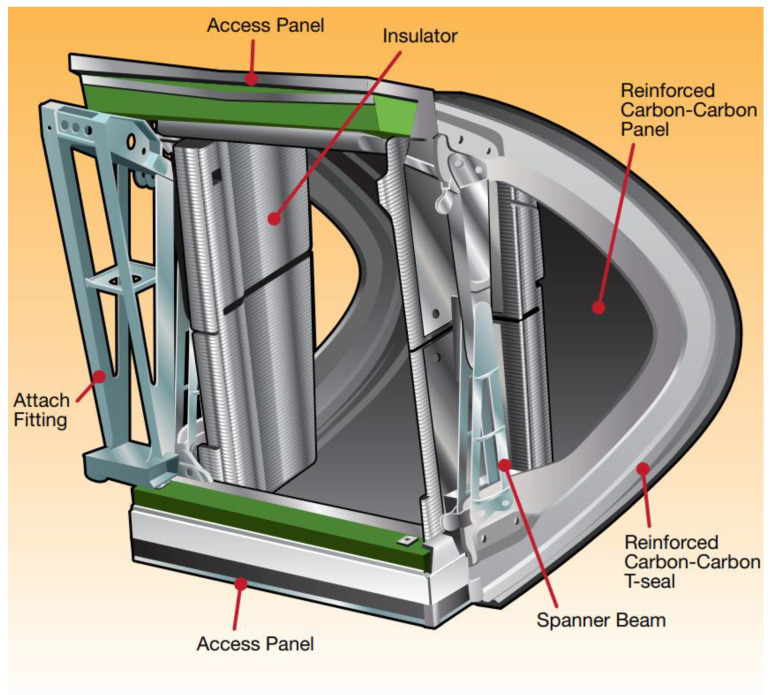
Thermal protection system of a space shuttle orbiter: tile placement (**top**) and wing panel assembly (**bottom**) [8]. Credit: NASA.

Reinforced carbon–carbon (RCC) is a composite material that was originally developed for the re-entry parts of intercontinental ballistic missiles (ICBMs). In space engineering, RCC is used for the thermal protection system (TPS) of re-entry vehicles for astronauts’ safe return to Earth. The use of RCC dates back to Space Shuttles. Prior to that, fiberglass honeycomb matrix infused with epoxy resin derived from phenols and formaldehyde was used for the ablative blunt-body heat shield of Apollo capsules [7]. Space Shuttles used RCC for their wings’ leading edge, nose cap and lower fuselage as depicted in Figure 1 [8]. Later, phenolic impregnated carbon ablator (PICA) was used in Stardust (1999), a mission to collect comet samples and bring them back to Earth. The Curiosity mission (Mars Science Laboratory, 2011) also adopted a phenolic impregnated carbon ablator for its rover-carrying spacecraft underwent entry, descent, and landing manoeuvres.

The Columbia Shuttle disaster happened during its Earth atmospheric re-entry in February 2003 after the RCC panels on the orbiter’s left wing were damaged during its launch and the compromised TPS went undetected throughout the mission. After the orbiter disintegration incident, the development of multi-phased composite systems followed. The constituents of a carbon–carbon composite system are matrices, fillers, and reinforcements [9]:

- Matrices: graphite, phenolic, and polyamides;
- Fillers: micro-filler (graphite, glass) and nano-filler (graphene nanoplatelet, carbon nano tube/fibre, silica);
- Reinforcements: glass fibre and continuous/chopped carbon fibre.

Note that presence of carbon nanotube or graphene nanofillers, even with small proportions at a few weight (wt) %, increases thermal conductivity significantly. This feature would not be desirable for TPS and offsets the advantage of improved mechanical properties by incorporating fillers into composites [10]. The linear increase in thermal conductivity in accordance of various filler contents is presented in Table 1, where thermoplastic polylactic acid (PLA, [−CHCO−]_n_) in its pure composition was compared with mixtures containing variational filler weight % of multi-walled carbon nanotubes (MWCNT) and graphene nanoplatelets (GNPs). The result shows that adding any kind of fillers increased the thermal conductivity compared with neat PLA, but the degree of increases differed amongst the filler compositions. For a given filler proportion (wt %), addition of GNP into PLA resulted in relatively high thermal conductivity values; compared with GNP-doped PLA, MWCNT-doped PLA measured only about half of the thermal conduction coefficients. Both thermal conductivity and thermal resistivity have their own applications and can be utilised effectively once the physical phenomena behind these experiment results are understood. The interfacial thermal resistance (Kapitza thermal flow resistance) is affected by the difference in dimensional geometries between the three-dimensional bulk polymer matrix and the nanofillers therein that can be one-dimensional (MWCNT) or two-dimensional (GNP). The two-dimensional filler forms strong networks and homogeneous dispersion within the bulk composite, leading to better thermal conduction than the one-dimensional filler. It is possible to predict a composite’s thermal characteristics affected by its matrix-filler morphology from molecular dynamics (MDs) simulations that calculates the phonon density/modes in two phases and the phonon transport in between [11]. This computation-aided design of simulations can replace or complement time-consuming design of experiments, especially for the new types of additionally manufactured composites whose design space would be larger than that of traditionally manufactured composites.

Besides filler selection, the use of functionally graded materials (FGMs) as reinforcements can be another design aspect to consider when it comes to the AM of TPS. The FGM layers or shells have gradually changing volume fractions along one or more dimensions of the solid. The idea of FGM was proposed in 1980s for thermal barrier applications [12]. Recently in the context of AM, reinforcements are usually fibrous materials whose different heating/curing techniques have been suggested, including capillary-, ultrasound-, or microwave-assisted approaches [13,14,15]. The FGM compositional gradient of SiC/C has a microstructure of sub-millimetre layers, from the pure SiC side to the pure C-C side with transitions, whose length scale is compatible with the AM resolutions. Both the SiC/C composite and the C/C composite reinforced with vapor-grown carbon fibre (VGCF) require high temperature (>1000 K) for oxidisation and carbonisation processes; as for pressure, these composites have a wide range of requirements ranging from several kPa to tens of MPa depending on fabrication steps. The AM settings for temperature and pressure are much less demanding if TPS printing materials are available; functionally graded nanocomposites of phenolic resin and carbon nanofiber can be fabricated, and the AM implementation of FGM would be possible in the near future [16,17]. Analytical methods and solutions have been developed for in-planar FGM problems to predict mechanical and heat conduction properties via finite element modelling (FEM), which is extendible to curved shapes theoretically and readily implementable in 3D-printers [16,18].

**Figure 2:**
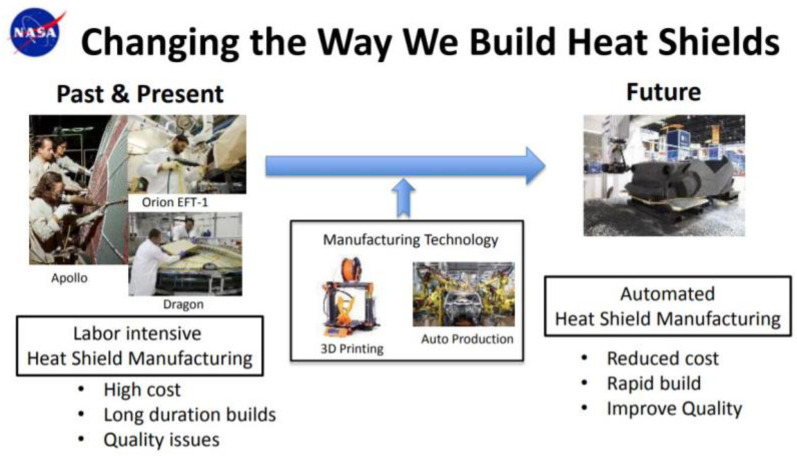
Thermal automation of manufacturing process of thermal protection systems with AM [18]. Credit: NASA.

The first technical demonstration of TPS additive manufacturing and assembly for space systems was made in 2019 when NASA Johnson Space Center and Oak Ridge National Laboratory investigated the feasibility of additively manufacturing heat shields with thermoplastics using a filament-based printer [19]. However, this method of Fused Deposition Modelling (FDM), also known as Fused Filament Fabrication (FFF), resulted in unstable char and swelling issues during a laboratory experiment mimicking atmospheric re-entry conditions. An alternative to this is thermoset resin mixtures and a customised commercial 3D printer which has an auger-based extruder. In this resin-based method, liquid-curable photopolymer is cured using a light source, whose optical-spot diameter determines the printing resolution [20]. As a result, the accuracy of this scheme is higher than that of FDM/FFF where the diameter and movement of a nozzle incur layer misalignment in x- and y-directions as well as layer squeezing and deformation in z-direction. The assembly of this ablative resin mixture was also successfully demonstrated by depositing them onto a miniaturised mock-up representing a sphere-cone-shaped re-entry capsule. Scaling up this TPS-AM process without gaps may remain a challenge, but the solution can be inspired from other disciplines such as the seamless, continuous-fibre AM of wind turbine blades recently solicited by the U.S. Department of Energy [21]. Above all, 3D printing of TPS has a potential for automating the present manufacturing approaches which are human labour intensive and prone to errors (Figure 2). Both fabrication and quality inspection will take much less time and effort compared with the Apollo era when epoxy resin had to be filled into each honeycomb cell by hand. The same benefit also applies to the maintenance of reusable launchers and suborbital aeroplanes.

### 2.2. Radiation Shields in Low-Earth Orbits or Deep Space

**Figure 3:**
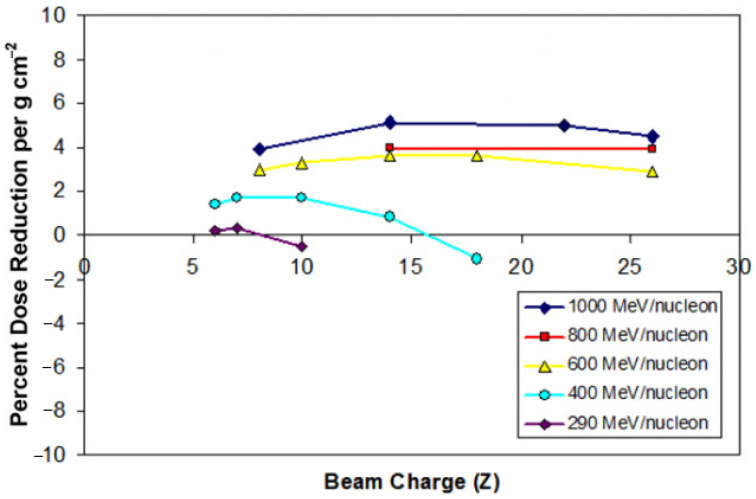
Percent dose reduction per areal density for the same shield (2.83 g cm^2^ polyethylene target) against different ion/energy combinations.

Due to the absence of Earth’s atmosphere and the surrounding magnetic fields, both robotic and crewed space vehicles in deep space are exposed to strong space radiation which is classified into ionising radiation and non-ionising radiation. The ionising radiation, because of its shorter wavelengths, has greater energy and higher possibility than non-ionising radiation to cause more damage to human DNA or microelectronics. Ionising radiation is subdivided into galactic cosmic ray (GCR) or solar particle event (SPE). Thought to originate from supernovae explosions, GCR contains high-energy nuclei (electron-free atoms) such as carbon, oxygen, magnesium, silicon, or iron (Fe^26+^). The heavy ions comprise only 2% of GCR, which are mostly protons and helium ions [22]; also called HZE meaning high (H) atomic number (Z) and energy (E), these heavy ions are difficult to shield against with current technologies. Polyethylene (PE, [−CH_2_−]_n_) was proposed as an alternative shielding material that absorbs more heavy iron ions than aluminium or lead. It was shown that PE shielding absorbed more 1 GeV/amu (atomic mass unit) ^56^Fe over a majority of ionization numbers (charges) than aluminium shielding in an experiment when similar areal densities were used (4.5 g/cm^2^ for PE and 5.0 g/cm^2^ for Al) [23]. In this setting, the ion charges were varied whilst maintaining the same level of incident energy consisting of iron ions. In another experiment, 19 different combinations of species and ions were used whose energy levels are shown in Figure 3. For lower energy levels, dose reduction effects were miniscule or even negative, as seen in Ne (10) for 290 MeV/nucleon and Ar (18) for 400 MeV/nucleon. For 600 MeV/nucleon, 800 MeV/nucleon, and 1000 MeV/nucleon, the dose reduction is more substantial and nearly invariant across element species as can be seen from horizontal plots. The percent dose reduction is 2–5% per g cm^2^ and roughly proportional to the shielding thickness, as demonstrated in a separate experiment where ~30% dose reduction against ^16^O was achieved using CH_2_ thickness up to 10 cm. Customarily, PE is used with other materials in the sleeping quarters for astronauts aboard the ISS, and other recent applications involve the use of ultra-high-molecular-weight polyethylene (UHMWPE) composites [24,25]. The shielding property of a material against GCR and secondary neutrons can further be enhanced as its hydrogen content exceeds that of PE (e.g., as a hydride, which is the anion of hydrogen) or is mixed with boron and nitrogen (e.g., borohydride or boronitride) [26,27].

The process of SPE accelerating solar energetic particles via solar flares (solar atmosphere) or coronal mass ejection (interplanetary space) is relatively easier to observe and considered to be more predictable than GCRs of an interstellar origin. A historical SPE database and prototype nowcasting/forecasting tools provide the frequency (or probability) of solar flare and SPE occurrences and their intensity [28,29]. Similar to GCR, protons from SPEs can be shielded in large space structures using water, food, clothing, and other materials rich in hydrogen, but these options are not always available in smaller spacecraft where UHMWPE-like materials can be used instead [23]. Solar flares also emit X-rays (0.1 to 0.8 nm in wavelength) whose ionisation effect can damage or unexpectedly change the characteristics of spacecraft electronics. In a ground experiment, the total dose and rate of X-rays were shown to alter the voltage curve of metal oxide semiconductor field effect transistors (MOSFET) that may lead to malfunctions in amplification or switching. The deviation of threshold voltage (V_th_) values was higher in p-type MOSFETs than n-types in an experiment of exposing CD4007 chips containing both types to 10-keV X-ray. The V_th_ or the ground-source bias where the drain current begins to increase changed from 1.48 V to 1.1~1.3 V for NMOS and from −1.31 V to −3~−4 V for PMOS with the 96 rad/s dose rate (Si) lasting 1 to 2 s, affecting their switching behaviours [30]. Although n-type MOSFETs exhibited smaller V_th_ deviations, they had more changes in the slope between the ground-source voltage and the drain current, leading to different amplifying characteristics. Compared with X-rays, irradiation effects from heavy ion beams (^35^Cl) were less marked, meaning that X-ray radiation shielding would be a higher priority for space electronics. Researchers demonstrated that polycarbonate–tungsten polymer matrix composite, 3D-printed with tungsten-loaded polycarbonate (PC) filaments, can increase the X-ray attenuation factor from 91 (rad/rad, pure PC) to 98 (rad/rad, 5% tungsten) at 40 keV and from 86 to 96 at 120 keV [31]. The volumetric loading and mass loading of tungsten microparticles were 0.3% and 5% here, respectively, which did not affect the other properties of pure PC such as mechanical and electromagnetic behaviours. Even 1% mass loading of tungsten increased the attenuation factor to 94 at 40 keV and 90 at 120 keV. Overall, these approaches may complement the existing, expensive radiation hardening techniques such as semiconductor chip shielding with borophosphosilicate glass (BPGS), silicon on insulator, silicon on sapphire, and so on [32].

**Figure 4:**
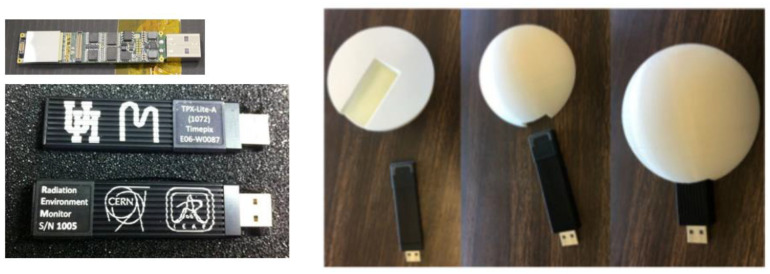
Radiation monitoring sensors and AM parts experimented inside the BEAM module [33]. Credit: NASA.

**Figure 5:**
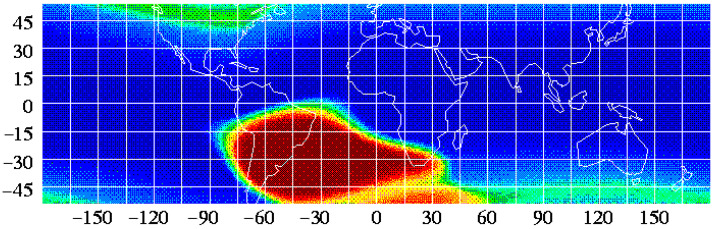
South Atlantic Anomaly (SAA, red area) [34]. Credit: NASA.

Acrylonitrile butadiene styrene (ABS, [−C_8_H_8_·C_4_H_6_·C_3_H_3_N−]_n_) is one of the first materials additively manufactured in orbit. Made In Space, a U.S. company, used its Additive Manufacturing Facility (AMF) installed on the International Space Station (ISS) to print and test radiation shields in 2017. In the tests, 3D-printed specimens with various thickness were attached to the inner walls of the Bigelow Expandable Activity Module (BEAM), an inflatable annexe to provide accommodation and workspace to astronauts [33]. Each hemisphere-shaped specimen housed a USB-like Radiation Environment Monitor (REM) sensor, shown in Figure 4, to measure the radiation levels within the hemisphere which was compared against those outside the hemisphere (still inside the BEAM) [34]. There were no noticeable changes in the radiation measurements for all plastic thicknesses (1.1 mm, 3.3 mm and 10 mm) in this experiment, however. The ISS and other satellites in low-Earth orbits pass through the South Atlantic Anomaly (SAA) where the Earth’s magnetic field has the local minimum, allowing X-ray and gamma-ray from solar flares to reach lower into the atmosphere and charged particles (protons) to be trapped therein [35,36] (Figure 5). The influence of SAA ranges from laptop crashes in Space Shuttles to fatal software errors including the one that ended the mission of Japan’s X-ray astronomy satellite in 2016 [37]. Back in 2012 during its cargo mission to the ISS, SpaceX’s Dragon spacecraft experienced a mild issue called Single Event Effect (SEE) which could be remedied by power cycling [38]. Astronauts passing through the SAA during their missions (Hubble, Mir, ISS) reported eye-related issues ranging from light flash (phosphene) to neuro-ocular syndromes [39]. Gamma ray shielding might be possible with bismuth-ABS filaments according to a nuclear medicine research, which requires further verification in the space environment [40]. It is noteworthy that the efficacy of composite shielding depends on the space weather conditions; solar maximum seasons are desirable for deep-space travels because solar activity deflects particles from distant galaxies (GCR) more efficiently [41]. A significant proportion of the GCR radiation consists of secondary radiation from neutrons and protons while the SPE radiation is attributed to primary particles. Secondary radiation is produced from nuclear reactions between the primary particles and the spacecraft shielding, propagating toward the spacecraft interior in a multipath/pass fashion. Shielding secondary neutrons is especially important as they may comprise 20% to 50% of the equivalent dose where the upper band represents thicker shield materials and longer spaceflight duration, ultimately limiting human spaceflight. Shielded quarters or wearable shields can be 3D printed using silicon, for example [42]. Research facilities in the Lunar Gateway in lunar orbits resembling the Additive Manufacturing Facility in the ISS orbiting the Earth, would be useful to develop AM technologies for deep space travel.

The FDM/FFF method and the light-curing approach mentioned in Section 2.1 (Heat Shields for Suborbital Flight), required the heat source and filament materials. Direct ink writing (DIW) is another interesting approach that can broaden the material and geometry choices with a variety of curing methods: ultraviolet, elevated temperature, and freeze drying to name a few [43]. Dimethylpolysiloxane (also called polydimethylsiloxane, (PDMS), [−Si(CH_3_)_2_O−]_n_), for example, exhibits neutron shielding properties after DIW using fillers such as boron, tungsten, tungsten (VI) oxide, or/and gadolinium (III) oxide. Initial results from the neutron radiography proved the shielding capability in all test cases except when silica (silicon oxide) was the filler. The ordering from the highest shielding specimen was: B^10^/Gd_2_O_3_ > B/Gd_2_O_3_ > B > Gd_2_O_3_ (>>SiO_2_ ≈ open beam without shielding) where B^10^ is a boron isotope. Other combinations reported in the medical sector for X-ray shielding, albeit not additively manufacturable yet, might take advantage of the DIW technique if the ink’s rheological constraints are met. Composite materials are regarded as a potential alternative to toxic lead aprons and non-lead aprons whose alloy sheets can be heavy. The alloy sheets contain non-lead heavy metals such as aluminium, tin, bismuth, tungsten, and titanium; both lead and non-lead metals are used in combination with polymer-based composites in radiation protective garments [44]. X-ray is a beam of high-energy photons induced from electron reactions and is shielded by lead-like materials dense in electron density, which is the main difference from high-energy particle (GCR) shielding using hydrogen-rich materials. Similar to these radiation protective garments, polyethylene terephthalate (PET, [−C_10_H_8_O_4_−]_n_) fibres with barium sulphate (BaSO_4_) provides lightweight, low-dose shielding for crews for aviation, which can extend to suborbital or space flights [45]. Zirconium, gadolinium, tantalum, lanthanum, and cerium are amongst other nanoparticle elements added as oxides to the polymer matrix for X-ray and gamma-ray shielding. The reporting results differ in terms of irradiation energy levels, from a few keV to hundreds of keV, which must be taken into account [46].

### 2.3. Issues in Printing and Assembling Structural Parts

There are several applications for 3D printing spacecraft structures with composites. After the printing of radiation shields, the Additive Manufacturing Facility (AMF) onboard the ISS proved compatible with 30 types of thermoplastics and polymers, producing over 200 parts and tools on orbit [47]. The AMF first used ABS and Green PE for manufacturing in zero gravity, the latter of which is a resin produced from sugarcanes [48]. Later, polyetherimide/polycarbonate (PEI/PC) was considered to produce tools used by astronauts during spacewalks. Unlike earlier 3D printable materials whose usage was limited to the interior of the ISS, PEI/PC has chemically stable properties adequate for exterior applications as well [49]; spare parts of the ISS were printed with PEI/PC due to its superior properties in harsh space environments including low outgassing in vacuum and durability against atomic oxygen or ultraviolet ray, to name a few. 

**Figure 6:**
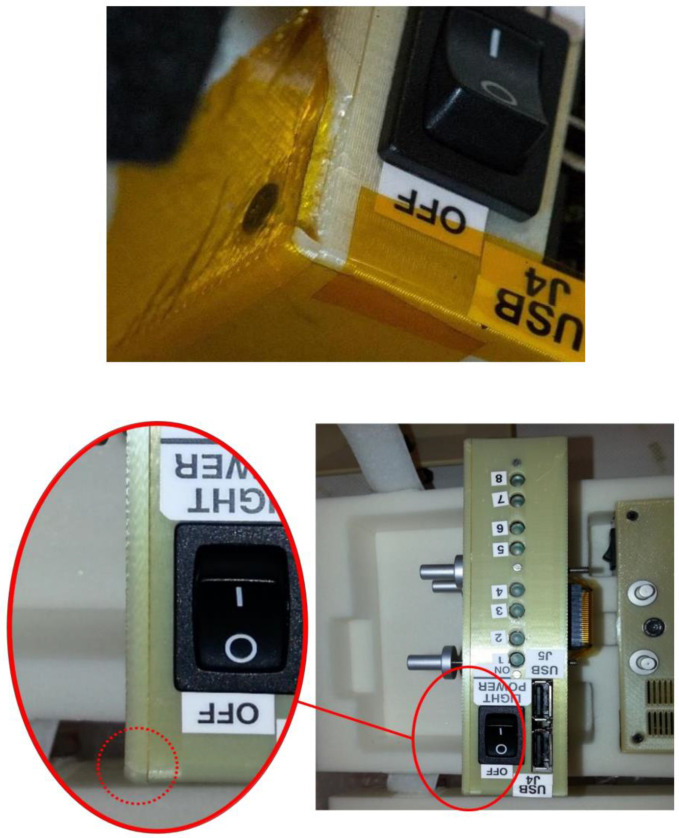
A broken 3D-printed part around the screw (top, onboard the ISS) and the same part during packaging for launch (bottom) [51]. Credit: NASA.

**Figure 7:**
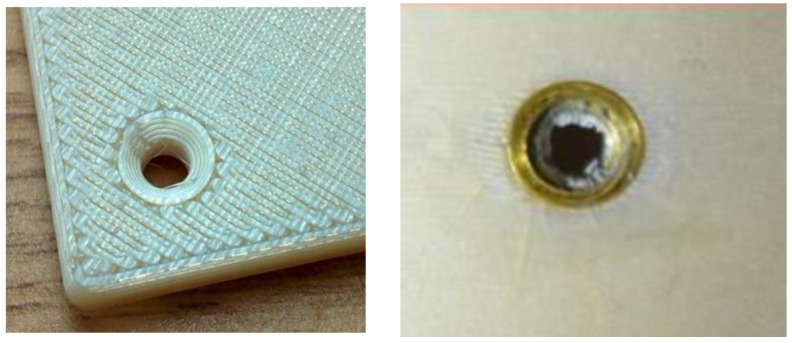
The threaded insert (**left**, avionics box) and radial cracks around it (**right**, specimen) [51]. Credit: NASA.

One of the PEI product family is the ULTEM^TM^ series; for example, ULTEM 9085 is amenable to 3D printing because its flow property is enhanced by incorporating the PC copolymer, leading to the PEI/PC denotation [50]. This material was used to print an experiment apparatus (Slosh avionics box) delivered to the ISS when a fracture was found at the corner, as shown in Figure 6 [51]. Follow-up investigations have revealed that the fracture did not develop during or after the launch, ruling out the launcher’s vibrations or crew handling from possible causes. One of the pictures taken during the pre-launch preparation indicated that a fracture existed when packed for launch, which was not evident to naked eyes under the given lighting conditions (Figure 6, right). Traversal forces due to the misalignment of joined parts or radial stresses from the over-torqued screw were amongst the possible causes of fracture. Instead of pan-headed (cylinder-shaped) or round (hemispherical) screws whose screwheads extrude from the surface, flat-head (inverted conical) screws were used to assemble the 3D-printed parts. Although flat-head screws save volume and prevent damage or injuries, they are susceptible to assembly errors in terms of both alignment and torque. This is because the conical head, sunk after fastening, cannot be checked easily whether it is under-torqued or over-torqued. The over-torqued scenario was re-enacted in a laboratory experiment in 2015, as shown in Figure 7, but direct comparisons are still not possible because the experimented specimen differed in the avionics box in geometries.

**Figure 8:**
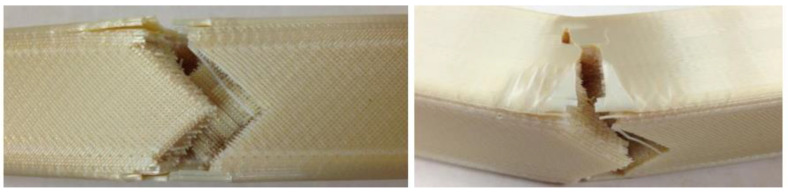
Gapped control sample that stayed together after the 3-point beam test, viewed from beneath (**left**, rastered bottom/top) and sideways (**right**, contoured sides) [51]. Credit: NASA.

On top of demonstrating the over-torqued scenario, the study investigated tensile/flexural behaviours with a design of experiments (DOEs). Different settings for printing and postprocessing (epoxy impregnation and surface sealing) were used. The extrusion paths were either solid (zero-inch gap or without airgap) or gapped (0.004” gap); five applicants in Table 2 were selected based on literature, among which Loctite 5110 is the least viscous and Hysol E-20HP is the tackiest [52,53,54,55]. Overall, gapped samples tended to have gentle break characteristics, as illustrated in in Figure 8, where the qualitative definitions of whether a break is ‘gentle’ or not is provided below. On the other hand, solid samples broke under a higher load but deflected less, indicating a higher flexural modulus (Table 2).

- Stayed together: the sample broke but was held together by a thin strand of material;
- Gentle break: the sample failed and fractured but did not separate into two pieces;
- Energetic break: the sample broke violently, impacting the walls of the test volume with considerable force;
- Unpredictable break: the type of break varied greatly from sample to sample.

Gapped samples yielded higher strain and lower stress than solid samples for both control and most of the experimented applicants. Extra spacing between adjacent extruding paths allowed for more deflection, but an average increase in mass and higher standard deviations were observed in gapped samples due to the lack of quality control. There are also other research works focusing on optimising several other printing parameters in addition to air gap; having a raster angle helps to reduce anisotropy because filaments can resist tensile loads in the length direction and the width direction of a sample. Similar rationale applies in the flexural modulus, as already shown by another NASA research [56]. More recent results, however, rather highlighted the role of other printing parameters; the most important were raster angle and raster width, followed by contour number and contour width, and the least important was air gap amongst five variables [57]. The role of air gap was less important for flexural strength contrary to its importance for tensile strength. The opposite phenomenon was observed in the other processing parameters, more influential to flexural strength but less influential to tensile strength, which requires further cross verification.

As discussed so far, the mechanical properties of AM parts are often unsatisfactory compared with the conventionally manufactured (i.e., injection moulding) counterparts due to their anisotropic nature. Even after in-planar isotropy is achieved, inter-layer adhesions (layer-stacking/building direction) are often weak. Instead of applicant postprocessing, pre-deposition heating with a laser was proposed by researchers who studied ULTEM ^TM^ 1010 in Table 3. Along with ULTEM ^TM^ 1000, ULTEM ^TM^ 1010 is the one of the strongest FDM/FFF printable materials possessing superior tensile strength, flexural strength and modulus compared with ULTEM ^TM^ 9085 [56,57,58,59]. The pre-laser heating scheme increased the build-direction tensile strength of ULTEM ^TM^ 1010 by 178%; the corresponding isotropy value was up to 82.8% which was defined as the strength ratio between the build direction and the build plane [60]. The maximum average tensile stress of 82.0 MPa is much closer to the manufacturer’s injection-moulded rating of 105 MPa after the 178% increase. The optimal laser power level that led to this result was found to be 1.6 W; the scanning electron microscopy (SEM) imagery of a fractured sample corroborates this isotropic-like behaviour, exhibiting fracture trajectories beyond the boundary of adjacent layers. The inter-layer interface is not distinguishable which suggests the healing phenomena at the microstructure near interfaces as well as the inter-layer interface. The power sweep between 0.3 W and 2 W was performed, and the tensile strength decreased at 2 W power level with burning and smoke. Because the degradation temperature is 510 °C, power levels equal to this or higher seem to generate local defects through decomposition during printing. When the power level is below 1.6 W, fewer entanglements occur between the two adjacent layers. The effect of laser or ultrasound treatments have been experimentally demonstrated, but there have not been many attempts for theoretical explanations, predictions, or verifications thereof [61]. Theoretical and experimental analysis of polymer film formation lead to optimising parameters for 3D printing processes either the manufacturing occurs in space or on Earth [62,63,64].

Polyether ether ketone (PEEK, C_19_H_14_F_2_O) is the final composite material to be introduced in this section. Both belonging to the polyaryletherketone (PAEK) family, PEEK and polyetheretherketoneketone (PEKK) have high stiffness and thermal resistance that distinguish them from PEI/PC despite their higher cost. PEKK is commonly found as either semi-crystalline or amorphous whilst PEEK may have a crystallinity level up to 40% [65]. The amorphous nature of PEKK facilitates inter-layer diffusion of polymer chains and layer adhesion, but PEEK has a higher continuous use temperature of PEEK (250 °C) than that of PEKK (150 °C, untreated) [66]. Because PEKK requires extra post-treatment to enhance its thermomechanical properties, it is used for 3D printing parts on the ground; PEKK is used in Boeing’s CST Starliner, a space taxi for transporting crews and cargo to and from the ISS, and is also speculated to have been used for astronaut helmets manufactured by SpaceX [67]. On the other hand, PEEK is compatible with the AMF on the ISS and is being considered by industries for in-space or off-Earth manufacturing in the future.

## 3. 3D-Printed Electronics

Printed electronics is a new research area for which electrically conductive or insulative thermoplastic filaments are needed. Additive manufacturing of reliable, high-performance circuitry is quintessential for internet or things (IoT) and electric-drive mobility for space/planetary exploration.

### 3.1. Traces and Substrates

Earlier 3D-printed electronics dates back to the printing and patterning of flexible microelectrodes that carry signals between microelectronic components [68]. Along with the nominal characteristics of conducting silver-ink traces, their failure modes due to excessive currents have been investigated [69]. Interconnects for an array of solar cells or light-emitting diodes (LEDs), have compactness comparable to satellite design. A wiring harness must be distributed under the tight constraints in terms of both volume and geometries, routed through the leftover space inside a satellite structure. Wiring harness distribution is non-trivial but is usually a low priority task because accommodation of the payload footprints (main functions) is considered first, and then the bus volume (supporting functions) is minimised before wiring is performed [70,71,72]. The nature of small satellite constraints often complicates the wiring process but is not any more facile in large satellites, either [73]. Embedded electronics with effective radiation shielding and thermal management would be useful for small satellites, robots, and astronaut tools. However, their all-in-one nature would make it difficult to repair anomalies or recycle after the end of life.

Because the holistic realisation of smart structures of a satellite has the aforementioned issues, research activities of 3D-printed wiring is more focused on component applications including circuits, sensors, and antennas. They consist of electrically conductive paths and components mounted on the non-conductive substrate, either or both of which can be additively manufactured. Table 4 shows various approaches of additively manufacturing embedded electronics, with arbitrary class numbering used here for the sake of convenience. For most electronics in either ground-based systems or space-based systems, both substrates and devices are commercially available via traditional manufacturing; both are marked “TM” in Class I of Table 4. As for the printed circuit board (PCB) substrates, flame resistant (FR)-4 composite, fabricated of woven fibreglass and epoxy resin, or glass fibre 10% (G10) have commonly been used in the National Electrical Manufacturers Association (NEMA) designation. Replacing these planar substrates with unusually or intricately shaped AM substrates provides extra degrees of freedom in functionalised design.

Copper traces on traditional PCBs conduct electricity with minimal losses because copper has an electrical resistivity as low as 1.6 × 10^−8^ Ωm (or 6 × 10^7^ S/m); this resistivity value is slightly higher than silver, but copper has a major advantage in cost effectiveness. Compared with etched copper patterns, copper-based inks are ten to one hundred times more resistive at a laboratory scale and a thousand times at commercial production levels [74,75]. Silver nanoparticle conductive inks have low viscosity (~10 cP) and thus are more adequate than copper conductive inks to be used in inkjet printers [76,77]. On the other hand, copper conductive inks are economical and mass producible, which has led to a wide variety of substrate applications including paper, fabrics, PET, PI, and HJT PV cells for high precision additive screen printing [78]. The combination of these non-PCB substrates or traditional PCBs and copper-printed paths may be considered as a hybrid manufactured substrate which is an intermediate stage between Class I and Class II [79]. Fabrication of more complicated circuit structures, such as double-sided or multi-layered, necessitates complete 3D-printed substrates (Class II). A study that tested several copper-mixed composites for soldering revealed that the tensile strength of substrate-component joints was the strongest for acrylonitrile butadiene styrene (ABS), closely followed by polystyrene (PS, [−C_8_H_8_−]_n_), and the weakest for polylactic acid (PLA) [80]. From the industry side, it is now possible to print a 10-layer PCB with densely populated components [81]. Stacking multiple layers of a PCB increases manufacturing costs of the traditional electronics industry but is still a valid choice if the user application necessitates a high number of circuit components per unit area while maintaining the PCB’s thickness and volume. A circuit designer can use interconnects through these layers or allocate multiple power and ground planes in the interior layers for enhancing electrical isolation. These advantages of multiple layers already exist in traditional PCBs, but 3D-printed PCBs might match or outsmart the traditionally manufactured counterparts in terms of electromagnetic compliance (EMC), power quality, and signal integrity by precisely controlling impedance of dielectric polymer ink [82]. Those extra layers can also be used for thermal management or other purposes, as briefly mentioned in the holistic approach. As for other ink materials, carbon black has higher electric resistivity than copper inks but can be used in harsh environments where metallic inks are prone to oxidation [83]. Polyetheretherketone (PEEK) filaments doped with carbon nanotubes (CNT) or/and graphite nanoplates were investigated in an ESA research initiative, where the conductive PEEK had conductivity of 10 S/m; a 100-fold increase from pure PEEK [84].

Conformal components for IT devices are immediate terrestrial applications of printed flexible PCBs [85]. An electronics printer will be launched to be used onboard the ISS, and radiofrequency components are being manufactured on the ground to be used on the ISS [86,87]. Even though the most conductive PEEK is too resistive to act as effective current paths, its thermal stability and thermoelectric property might be useful for (1) energy harvesting whereby waste heat is converted to electricity or (2) nuclear electric propulsion where heat is converted to electricity for ion propulsion.

### 3.2. Passive Components

Passive circuit components consume energy (resistor) and store electric energy in an electric (capacitor) or magnetic (inductor) field. In circuit design, resistors lower the voltage, in the form of a voltage divider, to match the required input voltage of other components; capacitors and inductors allow alternating current (AC) and direct current (DC) to flow through, respectively, whose characteristics are utilised in high-pass filters and low-pass filters in analogue circuits. Transducers are also classified as passive components because they convert physical signals to electrical signals without amplifying functions [88]. Table 5 summarises the electrical characteristics of copper foil, which is a conventional material choice for conducting paths on PCBs in the first row and those of passive circuit components in the second row [75,89,90].

These formulas are derived from physics and thereby the same regardless of whether a component is traditionally manufactured or 3D printed. The cross section of copper foil is usually rectangular, and circuit components have either circular (lumped) or rectangular (surface-mounted) cross sections. In Table 5, ρ is the resistivity, *l* is the length, *r* is the mean radius, *w* is the width of a foil or coil, and *n* is the number of turn (inductor coil) or stacks (capacitor); *A* stands for the cross-sectional area in a resistor and the overlapping area of stacks in a capacitor. The interconnects favour using copper because it has very low resistivity, resulting in negligible voltage drops despite micrometre-level thickness; for 3D-printed materials with a high resistivity value to be viable, the interconnect should have a large cross-sectional area or/and short length which will limit their applications to low-density circuitry. For passive components, on the other hand, AM may diversify existing two-dimensional geometries by printing unusual shapes or conformal patterns onto any curvilinear surfaces [91,92]. Researchers have been able to manufacture all basic circuit components including resistors (R), inductors (L), and capacitors (C) that can be combined to function as filters or antennas (LC tanks); these components along with conducting traces are mounted on the surface of a circuit board. Another conceivable design is buried channels or components, which are filled with the liquid metal paste or suspension; strictly speaking, the metal is not 3D printed, but the substrate bearing internal channels is. One downside is that the increased injection/flow resistance for channel widths less than 400 μm places a limit in manufacturability [93]. The influence of microgravity in space or decreased gravity on the planetary surfaces must also be assessed because the packing of liquid metal pastes into subsurface channels would be affected. The ground experiment reduces the paste’s conductivity by a factor of ten compared with the ideal paste; the influence of microgravity has not been researched yet. It would be worth investigating whether the reduced gravity and more uniform suspension of liquid metal is favourable to the electrical properties of buried channels or components. Another topic for future research would be fast and simple methods for improving the performance of AM circuit parts with conventional surface-mounted, extruding geometries; one-step electrodeposition proved to enhance the conductivity and other performance metrics (e.g., inductance for inductors) of 3D-printed electronic components [94].

Electrical machines or motors also benefit from composite 3D printing such as machine frame, shaft, and cooling structures that can be used for traction systems for next generation rovers [95,96]. An electric motor consists of a rotor and a stator which are attached to a shaft and a housing case, respectively. The stator, a hollow-cylindrical steel frame, is wound upright with copper wire for inductive properties [97]. Owing to the existence of copper winding, coolant layers are usually located around the motor housing and are referred to as a “water cooling jacket.” Similar housing-embedded channels have been prototyped for the 3D-printed motor of an electric motorcycle [98]. For this prototype to be realised, motor housing, traditionally fabricated of metal, needs to be 3D printed where thermosetting resins commonly used for injection moulding can be applied. It was claimed that composite housing for electric motors can achieve higher mechanical damping, corrosion resistance, and electrical performance than metallic housing (e.g., aluminium) [99]. 

Researchers at the Fraunhofer Institute for Chemical Technology and the Karlsruhe Institute of Technology (KIT) injection moulded a glass-fibre-reinforced thermoset (Vyncolit X7700) in their electric motor for traction applications [100]. Although they did not resort to AM, their choice of materials is easily transferable to AM. The above phenolic thermoset was chosen for housing due to its higher chemical stability than thermoplastics; it does not swell when in contact with water-glycol coolants or other automotive chemicals. Thermal conductivity of the material was not provided a high priority because the inside stator, not housing, can pump out heat. This novelty of their stator water circuit design can achieve reduced weight and increased power density. Table 6 compares indirect (conventional) and direct (proposed) cooling approaches. Their new direct-cooling scheme uses flat-section wires instead of round-section ones for conventional indirect cooling. The radial arrangement of flat wires leaves room for cooling channels within the stator, which is closer to the heat sources, i.e., copper winding of the stator itself or the inside rotor, rather than the housing where the water cooling jacket was located. For stator fabrication, the iron frame and copper winding were over-moulded using epoxy resin, EME-A730E, which contains aluminium oxide fillers to be thermally conductive with a conductivity value of 3 Wm^−1^ K^−1^. Mould cores are used to prevent the moulding process from filling the empty space where water channels should be. Lastly, the stator is assembled into the housing and fixed by connectors and union nuts.

Once AM is more widely used, the need for injection mould cores is eliminated, enabling more challenging channel geometries, such as internally finned tubes for stators or housings. It was reported that moderately thermally conductive carbon fibre-reinforced nylon reduced the copper winding temperature rise by 44% [101]. The integrative AM of mechanical parts and electric parts (PCB circuit) of a motor can be envisioned, as demonstrated using injection moulding during the KIT study. Other components of a tractive system, such as brake rotors, may also be 3D printed [102].

### 3.3. Active Components

In electronics, active components are defined as components that inject a net power into a circuit to perform various functions including signal manipulations, logical operations, optoelectronic displays, and so forth. The active components are usually semiconductor-based as seen in diodes, transistors, and integrated circuits (IC). Chip bonding and assembly thereof have mainly been investigated in the context of stretchable printed electronics. For ultra-thin wearable/flexible circuits, electrically conductive adhesives should be used for chip bonding because soldering is inappropriate. Ink stretchability and bending/pulling failure modes were analysed for a variety of combinations of silver flake inks (siloxane-. urethane-, polyester-based) and anisotropic conductive adhesives (epoxy-, acrylic-, silicone-based) [103]

Their most delicate variants such as programmable devices require microfabrication steps of photolithography, deposition, and etching whose nanometre-level resolution cannot be accomplished with AM; for example, a field programmable gate array (FPGA) is an IC consisting of hundreds of thousands of gates built on 28 nm or 14 nm process technologies [104,105]. Although the most advanced AM technology providing near-micron resolution would not achieve this level of complexity, a relatively simple microcontroller system-on-chip (SoC) prototype was created in 2017 by a group of researchers from Air Force Research Laboratory (AFRL) using silver-infused thermoplastic polyurethane for conductive traces [106,107]. This kind of flexible and stretchable SoC is to be adopted in a variety of internet of things (IoT) devices that personnel can wear whose target applications may include space activities. Since the SoC incorporates onboard memory and computing units, applications for soft robotics are also to be envisioned. 

Drop-on-demand inkjet printing is considered one of the most promising AM technologies for manufacturing transistors, diodes, and displays that are active components commonly used in integrated circuitry [108]. Small thickness of thin-film transistors (TFT) have made inkjet printing adequate for printing their vertically stacked structure. Printed logic gates, display driving circuits, and switching relays consist of an array of TFTs whose ink and source/drain (S/D) electrode films employ metal oxide, organic material, CNT, or graphene [109]. To address the failure concerns of TFT, restoration of interconnects thereof was considered by triggering a self-engineering process [110]. In this scheme, an inherently non-conductive liquid dispersion around the interconnect becomes electrically conductive as metallic nanoparticles therein respond to an electric field strength between 0.7 V/µm and 1.3 V/µm. Open faults of 20 µm lengths could be healed which corresponded to healing voltage values between 14 V and 26 V across them. The healed TFT interconnects withstood repeated bending of 100 cycles, and the self-healing technology was implemented in a TFT-integrated voltage amplifier. The IoT and industrial internet of things (IIOT) for space applications will make avail of other base technologies such as developing non-toxic inks and controlling the printing drop sequences [111,112].

## 4. 3D-Printed Devices for Life Support and Medical Purposes

Because medical assistance is not readily available in most space operations, it is essential to equip a spacesuit, space vehicle, or outpost with health monitoring capabilities for its wearer or crew. On top of passive health monitoring for prevention or diagnosis of disease and injury, telemedicine is desirable for crew medical care, providing necessary treatment and cure via remote or robotic means. However, medical and other life-supporting aspects complicate the logistics of crewed, long-duration missions as the mission planning should maintain a balance between supplies shortage and overstocking. If these life-critical supplies unexpectedly run out in the course of a deep space mission, restocking them from Earth is practically impossible. Carrying an excessive number of spares throughout the entire mission, however, will seriously degrade the feasibility and viability of the mission given the resource, space, and weight limits [113]. Printing medical and life-supporting supplies on demand reduces mission costs and contributes to the crews’ health and wellbeing. From a broader perspective, the holistically optimised logistics via 3D printing enhances the long-term sustainability of human space activities by minimising environmental impacts of the rocket launch such as carbon emission or debris generation.

Additive manufacturing for medical usage is an emerging area even for terrestrial applications. For example, electric conductive paths, discussed in Section 3, are also necessary in medical devices for data connection and transmission. Electrically conductive paths, USB cables, and USB-RS232 adapters fabricated of polyetheretherketone (PEEK) have been shown to achieve data transfer of 9600 bit/s (bps or baud) between two computers [114]. These data transfer interfaces support both wired and wireless communications; wired communications use adapters and wires between computers; wireless communication requires the same devices between the computer and an optical transmitter or receiver designed to transmit data to/from medical equipment [115]. For soft electronics whose conducting paths constitute sensors on human skins, multi-layer inkjet printing was applied to polydimethylsiloxane (PDMS) in conjunction with silver ink, the former of which mentioned in Section 2 for gamma ray shielding [116]. Incorporating 5G and IoT technologies will enable internet of medical things (IoMT) for prolonged human space activities.

The family of thermoplastics which polyetheretherketone (PEEK) belongs to is called polyaryletherketone (PAEK). Other plastics that belong to the PAEK family are: Polyetherketone (PEK), Polyetheretherketone (PEEK), Polyetherketoneketone (PEKK), Polyetheretherketoneketone (PEEKK), and Polyetherketoneetherketoneketone (PEKEKK or Ultrapek^®^). In the above acronyms, “E” and “K” represent ether (R–O–R’) and ketone (R2-C=O) groups, respectively, and R is the phenyl group (or phenyl ring) which is the simplest aryl. The wear performance of thermoplastic against itself was investigated for PEK, PEEK, and PEKEKK for spinal arthroplasty or small joints for the extremities in Ref. [117]. This biomedical application is relatively new and dominated by PEEK in terms of the market share although PEKEKK offers slightly higher melting temperature and tensile strength [118]. There are several commercial variants according to molecular weight and reinforcement fillers. Increased molecular weight leads to higher tensile elongation in unfilled PEEK. Filling glass fibre and carbon fibre increases flexural strength by 67% and 100%, respectively; and tensile/flexural modulus by 200% and 500%, respectively, compared with unfilled PEEK [119]. In a laboratory setting, PEKEKNK was synthesised by inserting a naphthyl group and was shown to have a higher rigidity and glass transition temperature then PEKEKK [120]. ESA and its industrial partners are developing a PEEK 3D printer operable in space and the prior ISS project (3D Printing in Zero G) demonstrated that microgravity did not generate significant engineering defects compared with the parts printed on the ground [113,121]. 

Whilst PAEKs’ medical usage is centred on surgical implants, medical/surgical instruments have been printed using less costly materials. Scalpel handles, sponge sticks, haemostats, forceps, and clamps are amongst the common handheld instruments used in various surgical procedures, which have been printed with acrylonitrile butadiene styrene (ABS) and evaluated by surgeons in practice [122]. As mentioned earlier, ABS has been used on the ISS for zero-gravity 3D printing to hundreds of spare parts. However, ABS printers should be operated in a sealed environment because of its ultrafine particle emission whose rates can be as large as 200 billion per minute [123]. Surgeons participating in the study also did not prefer polymer-based cutting tools, making them one of the cases where metal-polymer composite hybrid structures shall be used [124]. Possibly related research activities from the metallic printing side include the Aiming toward Zero Waste and Efficient Production of High-Tech Metal Products (AMAZE) initiative by ESA and electron beam freeform fabrication technology (EBF3) by the NASA Langley Research Center.

**Figure 9:**
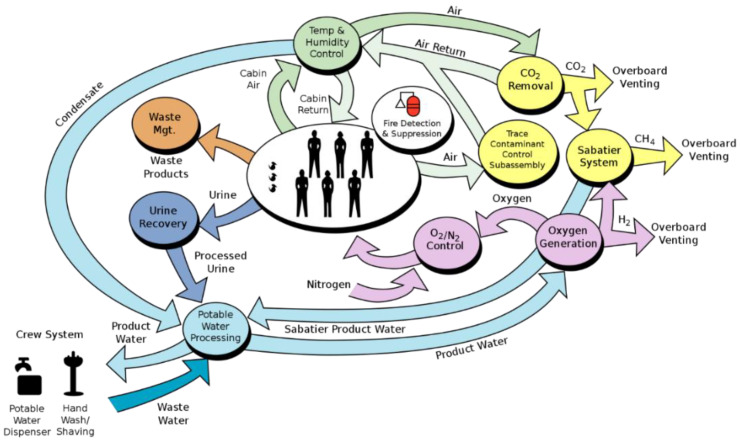
Environmental Control and Life Support Systems of the ISS [130]. Credit: NASA.

Polyarylether sulphones (PAES) are manufactured as membranes for haemodialysis/hemofiltration applications [125]. The PAES family consists of polysulfone (PSU), polyethersulfone (PES), and polyphenylene sulfone (PPSU), the last of which has better toughness than the first two in terms of elongation at break and impact strength [126]. Compared with their symmetric, homogeneously dense counterparts such as polyacrylonitrile and polymethylmethacrylate (PMMA), PAES membranes have asymmetric pores with directionality. In the PAES membrane installation, the larger pores facing upstream act as a prefilter which stops larger particles at the surface and allows smaller particles to enter [127]. Because the PAESs are steam sterilisable with high-temperature structural stability, they are used for food/beverage catering containers [128]; on account of the same advantage, they were also considered once for wastewater treatment apparatus in space-based Environmental Control and Life Support Systems (ECLSS) [129]. The application of PAES membranes was studied for different types of fuel cells (alkaline, proton exchange, etc.) which is another important part of the ECLSS (Figure 9) [130]. The commercialisation of PAES filaments and compatible printers will expedite their use in space where applicable.

Environment sensors, alongside with wearable sensors discussed in Section 4, can be used for ECLSS purposes and would benefit from AM. Ink materials such as palladium-silver, aluminium(-tin), and carbon–carbon–polymer composite are considered for the monitoring of NH_3_, CO_2_, CO, CH_4_, H_2_, humidity, temperature, and pressure [131]. Some of these environment-monitoring nano-sensors or medical instruments might be expendable, and the polymer recycler/refabricator is being developed for ULTEM^TM^ and other materials. The life-cycle properties, from a sustainability point of view, as well as the performance and safety of composite 3D-printed parts can be compared with those of metal 3D-printed ones obtained from the manufacturing and monitoring processes (e.g., metallography) of the ISS urine processor assembly, air filter, and scrubbers [132].

## 5. 3D-Printed Devices for Energy Applications

Electrochemical energy storage devices in terrestrial applications store (charge) and release (discharge) energy for a wide range of purposes in our life. Space applications include electric propulsion/drives in space or on planetary surfaces as well as high throughput payload operations [133,134,135,136]. The first AM component to fly in space was a custom-shaped battery box onboard a suborbital sounding rocket launched in 2013 [132]. As for human spaceflight, the Environmental Control and Life Support System (ECLSS) in Section 4 requires constant and backup power sources. Additive manufacturing technologies may enable on-ground preparation of unconventionally shaped devices or simple in situ repairs for which conventional manufacturing is not applicable for multiple reasons.

Volume or mass specific properties have been considered while choosing different battery types for space missions over time: energy density, cycle life, reliability, operating temperature, peak/continuous power, self-discharge rates, cost, manufacturability, etc., [137,138]. Currently, lithium-ion battery (LIB) technology is finding prominent use and replacing conventional battery technologies. The superior density characteristics of LIBs can be further enhanced by AM technologies such as complex 3D geometries in microscopic or macroscopic scales. For instance, LIB cells with 3D-printed electrodes have been shown to retain capacity of 85% even after operating at >10 C (charging/discharging the full capacity in 1/10 h) after thousands of cycles [139]. Interdigitated electrode for film, cube, or gyroid type electrodes and electrolyte composite achieve better power density and energy where the following four techniques are applicable [140]:

- Stereolithography (SLA): LLZ (Li_7_La_6_Zr_8_O_12_) for all solid-state battery electrolyte;
- Fusion deposition modelling (FDM): composite polymer electrolyte (CPE) and glassy carbon electrode (GCE);
- Direct ink writing: thick electrode and film electrode;
- Inkjet printing: lithium metal anode, air cathode, and sulphur cathode for lithium metal batteries.

The direct ink writing method is used for making a lithophilic host on which lithium metal is deposited in lithium metal anode cells whose shape is designed to withstand large volume changes [141]. In other electrode types, a hierarchically porous structure can be printed with FDM or SLA [142]. The hierarchically porous structure decreases the charge transfer distance and enables cells to achieve high power density and fast charging [138]. There have been attempts of printing electrolyte or patterning solid electrolyte interface ((SEI) between anode and electrolyte) of which the goal is to decrease the impedance. Solid polymer electrolyte or garnet type (Li_7_La_3_Zr_2_O_12_) solid electrolyte is printed with stencil printing, inkjet printing, and SLA [143,144,145]. Film electrodes are usually fabricated with the inkjet printing technique [146]. While a significant improvement in power and energy density characteristics have been achieved with AMT, further improvement in host material choice is needed for maintaining structural integrity and safety. Although precision material deposition can be achieved with finer needles, the clogging of nozzles used for material extrusion or inkjet printing limits the geometry choices, and needs rheological considerations.

Another notable class of energy storage devices is supercapacitors for self-powered electronics such as miniaturized sensors, biomedical implants, and portable RFID tags. Compared with batteries, supercapacitors have limited energy density but provide compelling power density. Micro-supercapacitors were inkjet printed with nanoparticle-based thin film nickel (II) oxide for highly conductive electrodes with magnesium perchlorate electrolyte to provide a wide voltage window [147,148]. A 3D-printed multiscale porous carbon aerogel (3D-MCA) achieved 6.5 times higher capacitance than the non-3D MCA, with a capacitance-per-mass density of 71.4 F/g at a high scan rate of 200 m/V [149]. Fabricated via direct inkjet writing and freeze-drying, the primary (500 μm) and secondary (nm-sized) cavities in the hierarchically porous structure increase the specific surface area up to 1750 m^2^/g [82,149]. The capacitance amounts to 148.6 F/g at −70 °C if the scan rate is maintained low (5 m/V), which can endure nights on Mars or the Moon with minimal heating.

Lastly, albeit non-power storing, photovoltaic panels can be 3D printed using polyethylene terephthalate (PET). The 200-micron-thick panel can be installed on nonplanar surfaces fabricated of plastic or steel. Whilst the PV panels can be fully recycled, their efficiency of generating electricity from sunlight is an order of magnitude lower than conventional ones. Unlike this example, most 3D-printed energy storage devices suffer difficulties in recycling. Both volume manufacture methods (FDM) and film manufacture methods (inkjet printing and direct ink writing) should consider assembly/disassembly aspects to address this issue.

## 6. Opportunities and Challenges

The advantages of AM for terrestrial applications can be translated into AM for aerospace applications: customization, functionalization, and consolidation (reduced part numbers). Space applications, in particular, have potentials to benefit from AM due to low volume, high complexity, and the criticality of lead times inherent in their products [150]. Composite AM technologies can be used in various areas of space applications, as already shown. Although fuse deposition modelling (FDM) and selective laser sintering (SLS) methods were mainly discussed here, a number of AM methods exist for polymer and other materials, as summarized in Table 7 [151,152]. Structural integrity is one of the common issues, and addressing it with reinforcement, replacement, or recycling/refabricating incurs extra mass, volume, and costs. Therefore, the performance gain and accompanying increases in mass, volume, and capital must be considered across a series of missions (a campaign), using a logistics model, rather than on a short-term basis [153,154]. In addition to the performance-cost trade-off, expediting the printing process is a challenge for large-sized parts where the resolution is also an issue. Even when the lead time is shorter than traditional manufacturing, due to the lack of flight heritage, prototyping/development processes including quality assurance can be challenging and time-consuming. Qualification of materials and products using ground-based experiments and simulation models (e.g., digital twins) are currently being investigated as possible solutions [150,154]. 

In addition to the aforementioned opportunities general to 3D-printed materials, there are a few areas of interest that are more specific to composites.

- Safety: oxygen compatibility of composites can be compared with metal components in propulsion or ECLSS systems where dust explosion is a significant safety hazard. The inflammability of composite and metal powders can be compared between AM and TM methods [155]. The impact of space debris from composite AM parts might also be of interest from a sustainability perspective [156];
- Miniaturized flight-testing platforms: the lack of gravitation affects the process and technology more than materials, whereas the vacuum has a significant effect on the material. Small satellites, sounding rockets, hyperbolic flights, or drop towers can be used to validate the effects of zero-gravity and vacuum, filling the gap between ground experiments and in-space manufacturing [157,158];
- Magnetic metamaterials: since composites do not interfere with magnetic fields, embedding magnetic materials endow stimuli-responsiveness, which may find uses for moving parts in robotics or enhanced treatment in biomedical applications [159,160].

## 7. Conclusions

This paper intended to provide a comprehensive review of applications where composite additive manufacturing can be adopted. Some of the structural applications have already flown in space, whilst other applications are more generic and experimental. With incomplete qualification standards for partially or fully 3D-printed products, the certification remains another significant challenge, on top of terrestrial challenges ever magnified in space. Nevertheless, there exists great potential for the long-term contribution of AM to sustainable space logistics. Technological road mapping and prioritisation can have synergetic effects if combined with more opportunities to access space or space-like environments (physical or virtual) and with artificial intelligence for process optimization [161]. For example, in-space additive repair as a permanent or temporary remedy can be an intermediate step on the road to in-space manufacturing [151].

## Figures and Tables

**Table 1:** Thermal conductivity of polylactic acid composites with varying filler contents (W/mK) [10].

Filler (wt %) ^1^	PLA + GNPs	PLA + MWCNTs	PLA + GNPs + MWCNTs ^2^
3.0	0.323	0.231	0.270
6.0	0.448	0.232	0.352
9.0	0.550	0.268	-
12.0	0.664	0.365	0.533

^1^ Filler 0% corresponds to neat PLA whose thermal conductivity is 0.183 W/mK; ^2^ wt % equal for GNP and MWCNT (1:1).

**Table 2:** Break characteristics, flexural modulus, and dimensions/mass change in samples according to applicant types and airgap configuration [51,52,53,54,55].

Applicant		Flexural Modulus		Mass/Dimensions	
Type	Viscosity	Solid	Airgap	Solid	Airgap
N/A (control)		Energetic (176 ksi)	Stayed together (117 ksi)	0% (control) 0/0 ^2^ μm	
Arathane 5750 A/B	100–250 ^1^	Energetic (189 ksi)	Gentle (113 ksi)	2.7% (103/49)	9.4% (141/−31)
Hysol E-20HP (hardener + resin)	5500–8000 40,000–90,000	Energetic (209 ksi)	Unpredictable (144 ksi)	3.6% (580/401)	3.6% (166/159)
Loctite 5110	36–66	Energetic		6.2%	6.4%
		(189 ksi)	(108 ksi)	(−28/10)	(−20/−2)
Probuild Marine	900–1100	Gentle		1.3%	4.3%
		(225 ksi)	(189 ksi)	(56/−46)	(37/−4)
BJB TC-1614	600	Energetic		10.6%	23.8%
		(333 ksi)	(306 ksi)	(44/34)	(49/0)

^1^ Viscosity units are in cps (centipoise), 1 cps = 0.001 Pa⋅s; ^2^ Contour/raster edges (both 1 inch = 2.54 cm initially).

**Table 3:** Mechanical properties of ULTEM series (units in MPa) [57,58,59,60].

	Ultem 9085				Ultem 1000		Ultem 1010	
	Injection Moulded (Sabic)	FDM Printed (Stratasys)	FDM (GRC *)	FDM (UiS **)	Injection Moulded (Sabic)	FDM + C (GRC *)	Injection Moulded (Sabic)	FDM Printed (UL ***)
Raster Angle		0°	±45°	0°		0°/±45°		
Tensile Strength	83	72	62	-	110	50/44	105	82
Tensile Modulus	3432	2200	2230	-	3579	2901 /2248	3200	-
Flexural Strength	137	115	92	127	165	-	160	-
Flexural Modulus	2913	2500	1901	2400	3511	-	3300	-

* GRC: NASA Glenn Research Center, ** UiS: University of Stavanger, *** UL: University of Louisville.

**Table 4:** Exemplary classification of electronics that are fully or partially 3D printed.

Class	I	II	III	IV
Substrate structure	TM	AM	AM (post-print)	AM (dual-print)
Embedded device	TM	TM	AM (pre-print)	AM (dual-print)

**Table 5:** Electrical properties of copper foil (rectangular) and passive components (assumed circular) [75,89,90].

	Resistance	Inductance	Capacitance
Copper Foil	$\frac{\rho l}{tw}$	$0.2l\left(ln\frac{2l}{w}+0.2235\frac{w}{l}+0.5\right)$ [μH] (*l* in mm, *t* negligible)	N/A
Circuit Components	$\frac{\rho l}{A}$	$\frac{\;r^{2}\;n^{2}}{8r+11w}$ [μH], Wheeler’s approximation (*r* and *w* in inches)	$n\frac{\;k\varepsilon_{0}A}{d}$

**Table 6:** Cooling techniques for copper winding inside electric motors (injection moulding).

	Inner		Outer
	Wire	Inter-Wire Space	
Indirect cooling	Round	Irregular and left empty	Embedded cooling sleeve
Direct cooling	Flat	Cooling channel with triangular section	Polymer housing without cooling sleeve

**Table 7:** Summary of AM methods and their characteristics [151,152].

Category	Types	Material	Advantages	Disadvantages
Material extrusion	Fuse deposition modelling (FDM)	Composite, Plastic	- Low temperature - Small equipment	- Weak strength * - Scalability
Vat (photo) polymerization	Stereolithography (SLA), Digital light processing (DLP),	Light resin (photo-	- Accuracy - Areal scalability	- Postprocessing - Weak to UV
	Cold DLP (CDLP)	polymer)		- Limited materials
Sheet lamination	Laminated object manufacturing (LOM)	Metal, Paper	- Areal scalability - Multiple materials	- Geometry choices - Post processability
	Ultrasonic consolidation (UC)			- Waste
Binder jetting	Powder bed and inkjet head (PBIH),	Metals, Polymers,	- Two materials (binder powder)	- Postprocessing - Weak strengths
	Plaster-based 3DP (PP)	Ceramics	− Speed, choices	− Accuracy
Material jetting	Material jet modelling (MJM), Drop on demand (DOD)	Waxes, Polymers	Accuracy and surface finishes	- Weak strengths - Print time
Power bed fusion	Selective laser sintering (SLS), Direct metal LS (DMLS), Electron beam melting (EBM)	Metals, Polymers	- Various materials - Recycling unused powder	- Energy use - Thermal distortion - Print time
Directed energy deposition	Laser metal deposition (LMS)	Metals, Polymers,	- Areal scalability - Speed, strength	- Capital cost - Resolution
		Ceramic	- Custom alloys	

* In the z-direction.

## Data Availability

Not applicable.
